# Pulmonary Embolism With Pulmonary Infarction in a Patient Using the Annovera® Segesterone Acetate and Ethinylestradiol Combined Vaginal Contraceptive Ring

**DOI:** 10.7759/cureus.63129

**Published:** 2024-06-25

**Authors:** Aveek Mukherjee, Sasmit Roy

**Affiliations:** 1 Pulmonary Critical Care, University of Tennessee Health Science Center, Memphis, USA; 2 Nephrology, University of Virginia, Lynchburg, USA; 3 Nephrology, Liberty University Medical School, Lynchburg, USA

**Keywords:** endocrine, combined hormonal contraceptive, vaginal contraceptive ring, contraception, annovera, ethinylestradiol, segesterone, pulmonary infarction, pulmonary embolism, thromboembolism

## Abstract

Combined hormonal contraceptives (CHC) are a very popular form of contraception among young women. Recently, vaginal contraceptive rings (VCR) have been formulated, offering greater convenience and ease of use. Venous thromboembolism (VTE) has been associated with CHC use and is a significant cause of mortality and morbidity in women. Here, we present the case of a 48-year-old woman who presented with right upper quadrant abdominal pain for four days associated with one day of shortness of breath. She had a history of anemia and abnormal uterine bleeding due to uterine fibroids. She was found to have a large embolus in the right pulmonary artery, associated with a right lower lobe pulmonary infarction. No evidence of lower-extremity deep venous thrombosis was found. She was using a segesterone acetate and ethinylestradiol combination VCR, which was removed. She was started on intravenous heparin anticoagulation with improvement in symptoms. This was later transitioned to an oral apixaban regimen prior to discharge. The exact mechanism of CHC-induced thrombotic risk remains unclear. They affect numerous proteins involved in the coagulation, anticoagulation, and thrombolytic pathways, thereby expressing their net thrombogenic potential. Estrogens have often been implicated as the more thrombogenic hormone, with progestogens being added to mitigate some of the risks. CHC use can cause a sixfold increased risk for VTE. Reducing the dose of estrogen and proper patient selection with attention to their risk profile remain essential for the safe use of these agents. This represents the first case report relating segesterone acetate and ethinylestradiol combination VCR to pulmonary embolism and infarction.

## Introduction

Hormonal contraception is a popular contraceptive used by young women [1]. Combined hormonal contraceptives (CHC) were initially only available in oral forms. Recently, vaginal contraceptive rings (VCR) have been formulated, with Nuvaring® (Organon USA, Inc., Roseland, NJ) being the first such approved in 2001 in the United States [2]. Another VCR formulation, Annovera® (TherapeuticsMD, Inc., Boca Raton, FL), was approved in the United States in 2018 [3]. Since the early 1960s, venous thromboembolism (VTE) has been recognized as a major hazard associated with CHC use [1,4]. The estimated annual incidence of VTE ranges from 104 to 183 per 100000 person-years among people of European ancestry, with an estimated worldwide burden of about 10 million cases per year [5,6]. The annual incidence of pulmonary embolism (PE) is estimated at 115 per 100000 persons in the United States, with an increasing trend globally [7]. PE carries a significant mortality burden of 4.1 deaths per 100000 women [7]. We present here a case of a non-fatal PE with pulmonary infarction in a 48-year-old woman using segesterone acetate and ethinylestradiol combination VCR.

## Case presentation

A 48-year-old nulliparous, non-menopausal woman presented to the emergency department complaining of right upper quadrant (RUQ) abdominal pain for four days and one day of shortness of breath. She had a history of anemia and abnormal uterine bleeding due to multiple uterine fibroids diagnosed a year prior. The abdominal pain started suddenly in the RUQ and was sharp, non-radiating, and worse with deep breaths. The shortness of breath was only due to exertion and was relieved promptly with rest. She did not report any cough, fever, chills, chest pain, hemoptysis, nausea, vomiting, bloating, or any obvious bleeding. On further inquiry, she reported the use of an Annovera VCR, starting a week prior to her symptoms. The patient was not using any other medications or supplements. She was a lifetime non-smoker without any personal or family history of thrombophilia or malignancy.

**Figure 1:**
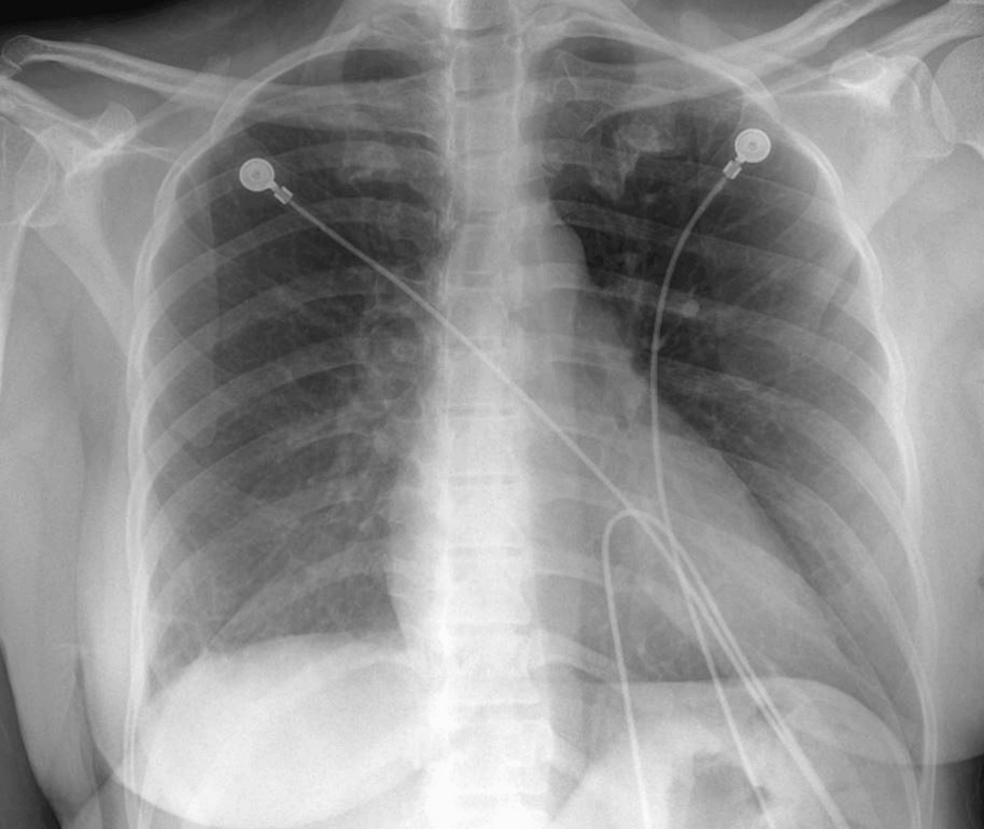
Normal chest X-ray

At initial presentation, her vitals were notable for a temperature of 99.6°F, tachycardia with a heart rate of 122 beats per minute, a blood pressure of 114/69 mm Hg, tachypnea with a respiratory rate of 22 breaths per minute, and a saturation of 99% on room air. A detailed physical examination was otherwise normal. Investigations were notable for anemia, with a hemoglobin of 7.6 gm/dL (reference 12.0-16.0 gm/dL), and neutrophilic leukocytosis, with a white blood cell count of 12300 cells/mm^3^ (reference 4000-11000 cells/mm^3^), and 84% neutrophils (reference 37-75%). Her complete metabolic profile was unremarkable. Procalcitonin was low at 0.07 ng/mL (reference <0.5 ng/mL), and a urine dipstick pregnancy test was negative. A chest X-ray was noted to be normal (Figure 1).

**Figure 2:**
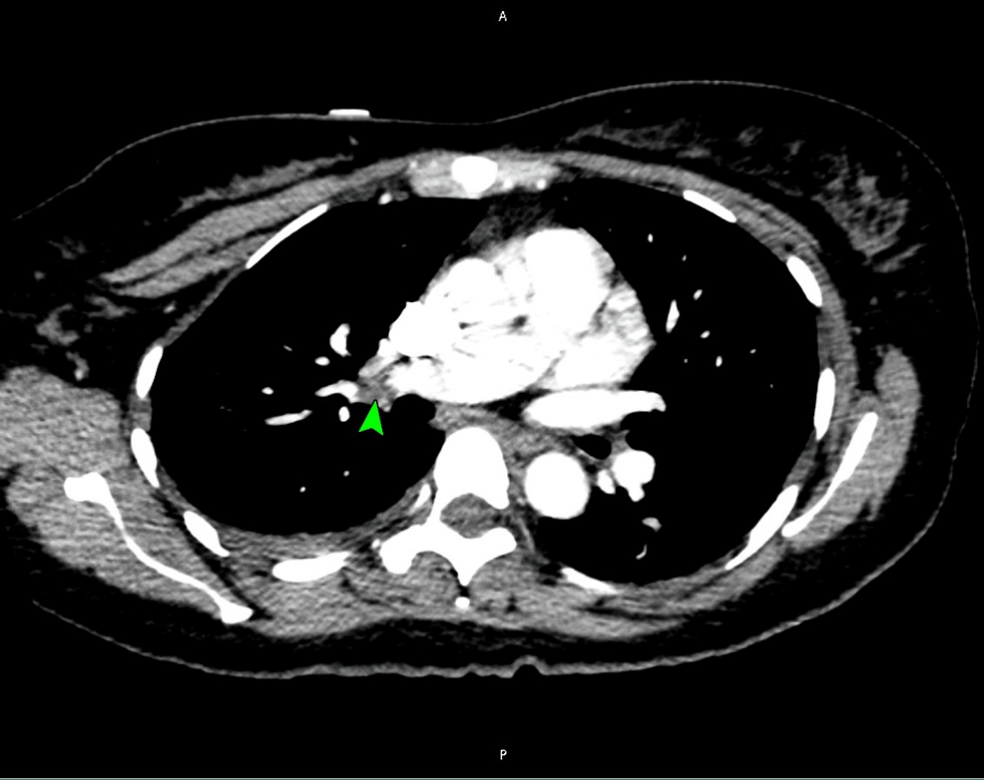
A CT pulmonary angiogram image showing a large pulmonary embolus in the right pulmonary artery (arrowhead) CT: computed tomography

**Figure 3:**
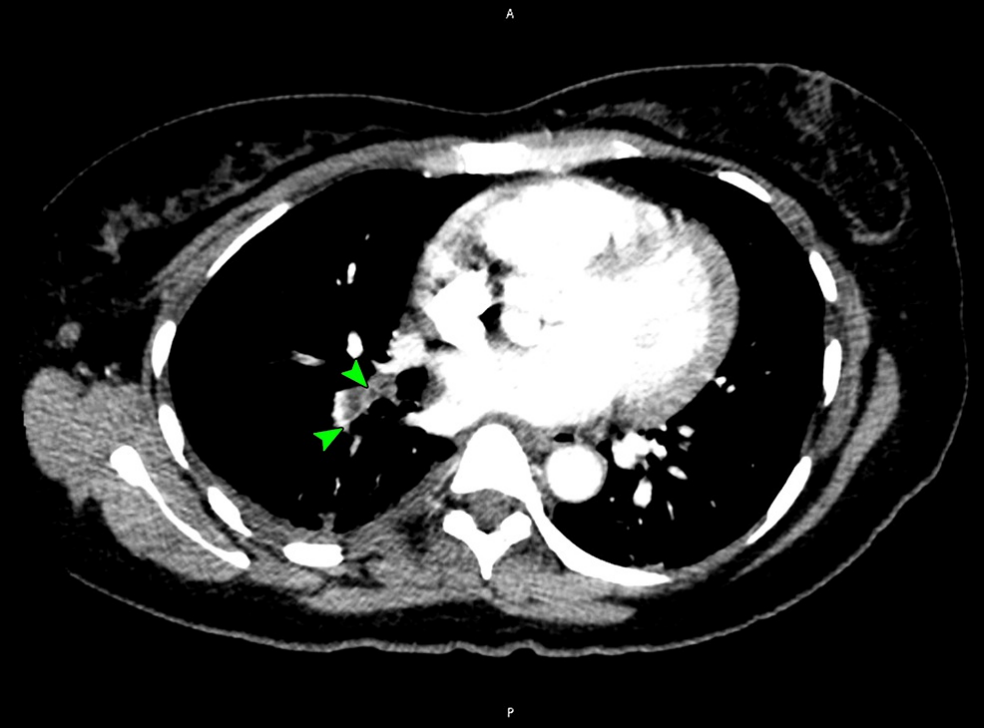
A CT pulmonary angiogram image showing a large right pulmonary embolus extending into the right lower lobe segmental branches (arrowheads) CT: computed tomography

A duplex venous ultrasound of her lower extremities was unrevealing. A CT pulmonary angiogram revealed a large pulmonary embolus in the right pulmonary artery (Figure 2), extending into the right lower lobe segmental branches (Figure 3).

**Figure 4:**
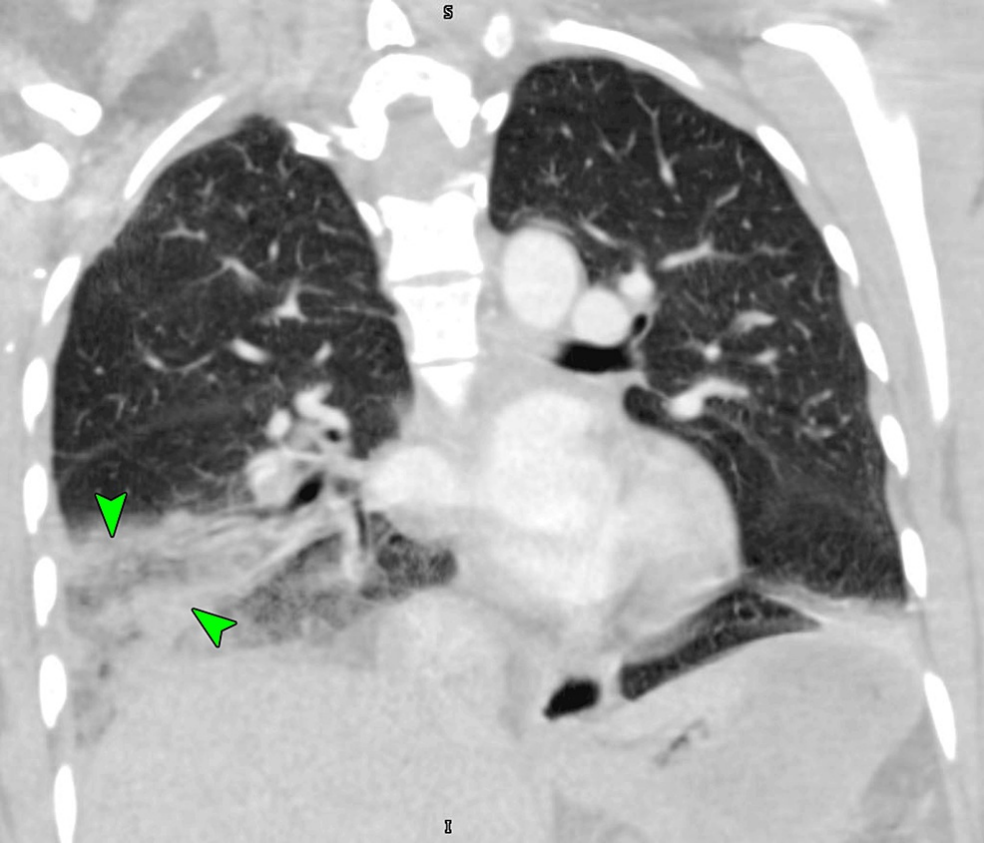
A CT pulmonary angiogram image showing a wedge shaped area of consolidation noted in the right lower lobe, representing a pulmonary infarct (arrowheads) CT: computed tomography

There was also a roughly triangular area of consolidation noted in the right lower lobe (Figure 4), which likely represented a pulmonary infarction, leading to pleuritic chest pain.

With a Naranjo score of seven, the PE and pulmonary infarct were considered probable adverse reactions to the VCR. The patient was admitted and started on an intravenous heparin drip for therapeutic anticoagulation. The VCR was promptly removed. Screening for factor V Leiden mutation, anti-thrombin III deficiency, prothrombin gene mutation, protein C deficiency, protein S deficiency, and antiphospholipid antibodies was normal. An echocardiogram was found to be normal. Analgesics were administered for her RUQ pain, which rapidly abated. She was transitioned to oral apixaban, educated about the possible use of progestin-only contraceptives, and discharged. Unfortunately, the patient was lost to follow-up after discharge.

## Discussion

The CHCs have long played a central role in empowering women to take control of and maintain their reproductive health. However, VTE associated with CHC use has been a constant concern due to its widespread adoption. Apart from VTE, CHCs have also been implicated in cerebral venous sinus thrombosis and stroke [8-10]. Mesenteric venous thrombosis, retinal vein occlusion, and myocardial infarctions have been reported as well [11].

The exact mechanism(s) of CHC-induced increased thrombotic risk is unclear [4,11]. One major mechanism may be altered levels of numerous hepatically synthesized proteins. Historically, the estrogenic component of the CHC has long been considered to be more thrombogenic [4,11]. Its effect on platelet number and function is unclear, but it does upregulate the von Willebrand factor expression [11]. Estrogens increase plasma levels of factors II, VII, VIII, X, and fibrinogen but decrease factor V levels and tissue factor pathway inhibitors [4,11]. It also decreases antithrombin and protein S and induces an overall resistance to activated protein C [4,11]. Estrogens may also upregulate levels of thrombin-activated fibrinolysis inhibitor, which is an independent risk factor for venous thrombosis [4]. However, the net effect on fibrinolysis may be equivocal [4,11]. Progestogens have been traditionally added to CHCs on the theoretical premise that, on their own, they are not thrombogenic, and when added to estrogens, they may favorably modify the thrombogenic risk [12]. Moreover, the progestogens may have a lower risk of arterial thrombosis due to their beneficial effects on high-density lipoprotein cholesterol [4]. However, all these estrogen-related changes are more pronounced in combination with a third-generation progestogen containing CHC, especially desogestrel and gestodene [4,11,13]. These effects of estrogen were considered to be dose-dependent, and hence the modern CHCs are available with a reduced estrogen dose, though the benefits of reducing VTE may be limited [4,11]. The data is conflicting regarding which route of CHC drug delivery confers a greater VTE risk when comparing transdermal, oral, or vaginal routes [11,14]. In summary, the upregulation of procoagulant effects and downregulation of anticoagulant effects, combined with an equivocal effect on fibrinolysis, induces a net prothrombotic state when using CHCs.

Segesterone acetate is a potent fourth-generation progestogen without any androgenic, estrogenic, or glucocorticoid activity [3,15]. It is inactive orally but efficiently absorbed by the vaginal mucosa [3]. The segesterone acetate and ethinylestradiol combination VCR is an effective, unique, and convenient contraceptive as it can be reused for a whole year [3]. With this combination, it was noted that the plasma levels of factor VIII, fibrinogen, and protein S were elevated from baseline but still within the normal range [16]. This could suggest a less thrombogenic profile compared to other CHC combinations. However, the effect of the combination on sex hormone-binding globulin (SHBG) remains unclear. One study noted no change in estrogen-associated changes in plasma SHBG when comparing oral and vaginal drug delivery [17]. Another study noted an elevation of plasma SHBG above normal, especially in patients who weren’t exposed to CHCs before [16]. Though VTEs and cerebral venous thrombosis were noted to occur with this combination during initial studies, the risk was similar to other vaginal and transdermal CHC preparations [3,15]. It was suspected that a BMI >29.0 kg/m^2^ could be a risk factor for VTE events with this CHC [15].

VTE remains a major clinical burden worldwide and a significant cause of mortality and morbidity [5-7,18]. In 1856, Rudolf Virchow identified the factors (Virchow’s triad) that lead to thrombosis: hypercoagulability, stasis of blood flow, and vascular endothelial damage [6,18]. There may also be inherited and acquired risk factors that increase the risk of VTE in the affected populations [6]. The inherited factor V Leiden, prothrombin gene mutation (G20210-A), antithrombin deficiency, protein C deficiency, and protein S deficiency may confer a five- to 10-fold increase in VTE risk [6]. Factor V Leiden especially increases the risk of VTEs with concurrent CHC use by a factor of 35 [4]. Notably, estrogen-containing CHC use may independently cause a three- to six-fold increase in VTE risk [4,6,13]. The risk of VTE from CHC use is highest in the first six months [11]. Apart from the above-noted reasons for increased VTE with CHC use, VCR may have another unique risk factor associated with its use. Due to the ease of VCR use, the patient may not recall being on a medication, which may significantly increase the risk of adverse events [19]. Over the last decade, multiple studies have reported either a similar or slightly higher risk of VTE with VCR use compared to other CHC formulations [20-22]. As noted previously, an individual’s underlying thrombophilia may greatly elevate the risk of VTE with VCR use [15,20,22]. However, overall, it appears that the risk of VTE with VCR use may be no different than other CHC formulations. More research is needed to clarify this [22]. Most PEs originate as deep vein thrombi in the lower extremities, detach from the original site, and are later lodged in the pulmonary arterial system after traveling through the systemic veins and through the right-sided heart chambers [6]. Depending on the location, size, and number of emboli, hemodynamic consequences including right heart dysfunction, hypotension, hypoxia, and even death may ensue [6]. VTE is the etiology of about two-thirds of pulmonary infarctions [18]. The lungs have dual blood supply via the bronchial (oxygenated) and pulmonary (deoxygenated) arteries [18]. Due to the absence of local bronchopulmonary anastomoses, the occlusion of the distal pulmonary artery results in a hemorrhagic infarct of the pulmonary tissue due to the extravasation of blood from the bronchial vessels [18]. This may irritate the pleura, causing pleuritic pain, which may occasionally present as RUQ pain, as in our patient [18]. These infarcts may appear as wedge-shaped, broad pleural-based densities with their apex toward the hilum on a computed tomogram. Occasionally, there may be an internal lucency (suggesting necrosis) with an enlarged vessel at the apex (feeding vessel sign) [18].

In our patient, due to the possibility of an adverse reaction to the VCR leading to PE and infarction, we utilized the Naranjo algorithm to determine such a risk [23]. With a score of seven, an adverse drug reaction was considered very probable. Our patient was a good candidate for a CHC according to both the World Health Organization and the Centers for Disease Control and Prevention criteria, with advantages outweighing the risks of VCR use [24,25]. However, though progestin-only hormonal contraception remained an option for our patient, the risk of further thromboses in patients with a history of VTE with CHC use remains unclear [1,4,11,12]. Finally, hysterectomy remained another option without the risk of VTE if the patient was agreeable.

## Conclusions

CHCs are convenient and widely used; however, their adverse effects may be life-threatening. Proper patient selection with close attention to individual risk factors is likely key to their safe use. To our knowledge, this represents the first published case relating segesterone acetate and ethinylestradiol combination VCR to PE and infarction. This is a relatively new VCR formulation, and we suggest keen post-marketing surveillance to reduce adverse outcomes for patients.
